# Circulating linoleic acid and alpha-linolenic acid and glucose metabolism: the Hoorn Study

**DOI:** 10.1007/s00394-016-1261-6

**Published:** 2016-07-14

**Authors:** Mieke Cabout, Marjan Alssema, Giel Nijpels, Coen D. A. Stehouwer, Peter L. Zock, Ingeborg A. Brouwer, Amany K. Elshorbagy, Helga Refsum, Jacqueline M. Dekker

**Affiliations:** 1 Department of Health Sciences and EMGO Institute for Health and Care Research, Vrije Universiteit Amsterdam, Amsterdam, The Netherlands; 2 Unilever Research and Development, Vlaardingen, The Netherlands; 3 Department of Epidemiology and Biostatistics and EMGO Institute for Health and Care Research, VU University Medical Center, Amsterdam, The Netherlands; 4 Department of General Practice and EMGO Institute for Health and Care Research, VU University Medical Center, Amsterdam, The Netherlands; 5 Department of Internal Medicine and Cardiovascular Research Institute Maastricht (CARIM), Maastricht University Medical Centre, Maastricht, The Netherlands; 6 Institute of Basic Medical Sciences, Department of Nutrition, University of Oslo, Oslo, Norway; 7 Department of Pharmacology, University of Oxford, Oxford, UK

**Keywords:** Serum fatty acids, Linoleic acid, Alpha-linolenic acid, Type 2 diabetes, Glucose, Hemoglobin A1c

## Abstract

**Purpose:**

Data on the relation between linoleic acid (LA) and alpha-linolenic acid (ALA) and type 2 diabetes mellitus (T2DM) risk are scarce and inconsistent. The aim of this study was to investigate the association of serum LA and ALA with fasting and 2 h post-load plasma glucose and glycated hemoglobin (HbA1c).

**Method:**

This study included 667 participants from third examination (2000) of the population-based Hoorn study in which individuals with glucose intolerance were overrepresented. Fatty acid profiles in serum total lipids were measured at baseline, in 2000. Diabetes risk markers were measured at baseline and follow-up in 2008. Linear regression models were used in cross-sectional and prospective analyses.

**Results:**

In cross-sectional analyses (*n* = 667), serum LA was inversely associated with plasma glucose, both in fasting conditions (*B* = −0.024 [−0.045, −0.002]) and 2 h after glucose tolerance test (*B* = −0.099 [−0.158, −0.039]), but not with HbA1c (*B* = 0.000 [−0.014, 0.013]), after adjustment for relevant factors. In prospective analyses (*n* = 257), serum LA was not associated with fasting (*B* = 0.003 [−0.019, 0.025]) or post-load glucose (*B* = −0.026 [−0.100, 0.049]). Furthermore, no significant associations were found between serum ALA and glucose metabolism in cross-sectional or prospective analyses.

**Conclusions:**

In this study, serum LA was inversely associated with fasting and post-load glucose in cross-sectional, but not in prospective analyses. Further studies are needed to elucidate the exact role of serum LA and ALA levels and dietary polyunsaturated fatty acids in glucose metabolism.

**Electronic supplementary material:**

The online version of this article (doi:10.1007/s00394-016-1261-6) contains supplementary material, which is available to authorized users.

## Introduction

Dietary fatty acids affect blood lipid concentrations [1] and are related to the risk of developing coronary heart disease (CHD) [2]. Therefore, most nutrition recommendations advise to limit intakes of saturated fatty acids (SFA) and trans fats and replace these with unsaturated fatty acids, in particular polyunsaturated fatty acids (PUFA) for optimal CHD risk reduction, which in the diet mainly occur as omega-6 linoleic acid (LA) and omega-3 alpha-linoleic acid (ALA) [3–5].

Type 2 diabetes mellitus (T2DM) is a rapidly growing chronic metabolic disease [6] that not only confers a high risk of complications, but also puts a heavy burden on patients’ quality of life and on healthcare resources. Data on the role of dietary fatty acid intake, in particular on the plant-based PUFA linoleic acid (LA) and alpha-linolenic acid (ALA), in the development of T2DM are limited. According to FAO/WHO guidelines, there is probable but inconclusive evidence that increasing LA lowers the risk of developing T2DM [7]. Some prospective cohorts reported that higher dietary LA intake is associated with lower T2DM risk [8–10], while others showed no significant associations [11–13] or even a positive association [14].

Dietary intake is often difficult to determine due to the limited accuracy of the measurement and the food composition tables, and the tendency to underestimate total energy intake including fat intake [15, 16]. The intake of the nonendogenously produced fatty acids LA and ALA can also be assessed more objectively via measurement of circulating fatty acids in serum, plasma or erythrocytes. These levels provide a relatively accurate reflection of the dietary fatty acid intake [17] over past several weeks or months [18]. The association of fatty acids in serum, but more often in plasma or erythrocytes, with T2DM has been investigated in a few prospective studies. Most researchers observed that higher proportions of LA were associated with a significantly lower T2DM risk [11, 13, 19–22], but others found no association [23]. A recent systematic review on the omega-3 fatty acids ALA, eicosapentaenoic acid (EPA), and docosahexaenoic acid (DHA), including six cohorts, concluded that higher circulating ALA and not EPA or DHA was associated with a nonsignificant trend toward lower risk of T2DM [24].

Researchers that studied both the fatty acid intake from diet and fatty acids in the circulation concluded that associations with diabetes risk were stronger with the use of circulating LA and ALA blood levels than with dietary intakes assessed by food frequency questionnaire (FFQ) [11, 13, 14]. Most earlier studies on these fatty acids and diabetes incidence relied on diagnosed, self-reported diabetes which is an underestimation of the total burden of diabetes. It is estimated that worldwide, half of the diabetes patients are undiagnosed [6]. In the present study, all current markers used to diagnose T2DM were measured [11]. In this data set, we investigated the cross-sectional and the prospective associations of serum LA or ALA with fasting plasma glucose (FPG), post-load glucose (PLG), and glycated hemoglobin (HbA1c).

## Method

### Study population

The Hoorn study is an observational cohort study on glucose metabolism in the general Dutch Caucasian population which included 2484 participants, aged 50–74 years at study initiation in 1989. The detailed research design and methods of this initial Hoorn Study are described elsewhere [25]. For the examination cycle in 2000–2001, an invitation was sent to all surviving patients with T2DM (*n* = 176) and random samples of individuals with impaired glucose tolerance (*n* = 193) and normal glucose tolerance (*n* = 705), based on their glucose tolerance status at the 1996–1998 examination. Of all invited participants, 648 (60 %) participated in this study [26]. In addition, 195 (90 %) of the 217 newly diagnosed patients with T2DM from Hoorn screening study (1998–2000) were included, as well as 60 diabetes patients, newly diagnosed in general practice [27]. We excluded people taking oral glucose lowering medication (*n* = 48), patients with known diabetes at previous Hoorn study examinations (*n* = 19), people with missing information on diabetes status (*n* = 15), and people with missing information on serum fatty acids (*n* = 154). In total, 667 individuals were included in the current cross-sectional analyses. Of the *n* = 667 participants at baseline, *n* = 417 participated in the follow-up examination performed in 2008, where they underwent an interview to obtain general and medical history-related information, a physical examination, and analysis of blood samples [28]. For the prospective analysis, we additionally excluded all baseline T2DM patients (*n* = 160) because treatment initiation may have impacted on the outcomes. The 257 individuals were included in the current prospective analyses. This study was conducted according to the guidelines laid down in the Declaration of Helsinki, and all procedures involving human participants were approved by the VU University Medical Centre Ethics Committee. Written informed consent was obtained from all participants.

### Fatty acid profile

A fasting blood sample was drawn for the determination of serum total lipids at baseline. The VU Medical Center stored the serum samples for ~10 years at −80 °C. Fatty acid profiles in serum total lipids were assayed by gas liquid chromatography with flame ionization detection at AS Vitas, Oslo Innovation Center, Oslo, Norway. Serum samples, thawed in fridge overnight, were vortexed, centrifuged, and transferred into new vials. Internal standard (triheptadecanoin) was added, and fatty acids were methylated into methyl esters (FAMEs) with MeOH HCl. The FAMEs were extracted with hexane. After neutralization with KOH in water, mixing and centrifuging, the hexane phase was injected into the GC-FID. Analysis was performed on an Agilent 7890A GC with a splitless injector, a 7683B automatic liquid sampler, and flame ionization detection (Agilent Technologies, Palo Alto, CA, USA). Separations were obtained using a SP-2380 column (30 m × 0.25 mm i.d. × 0.25 µm film thickness) from Supelco (Bellafonte, PA, USA). FAMEs were identified by comparison with known standards. An external standard containing known amounts of relevant FAMEs (Supelco 37 component FAME Mix) was included in each run to correct for differences in fatty acid response factors. Fatty acid content was calculated based on the area % of peaks and response factors relative to 18:0. The method has been validated for quantification of fatty acids according to US FDA Guidance Document on Bioanalytical Method Validation. The inter-assay coefficient of variation varied between 2 and 4 %.

### Glucose levels

FPG levels were determined in serum by a hexokinase method (Roche Diagnostics GmbH, Mannheim, Germany), and samples for PLG were taken 2 h after ingestion of a standard 75-g oral glucose tolerance test at both examinations. The HbA1c result at baseline was calculated as a ratio to hemoglobin by an ion-exchange high-performance liquid chromatography (HPLC) based on the separation on a Mono-S column (Pharmacia, Uppsala, Sweden). At follow-up HbA1c was measured by a turbidimetric inhibition immunoassay. The Diabetes Control and Complications Trial (DCCT) standardization of HbA1c was only present in the follow-up measurements, so an internal validation between HbA1c measurements and DCCT standardization methods was needed. Conformity between the two HbA1c measuring methods was found that disputes a significant impact of the dissimilar laboratory methods on our outcomes [29, 30]. Glucose tolerance status at baseline was determined according to the most recent WHO criteria [31, 32]. FPG (≥7.0 mmol/L), PLG (≥11.1 mmol/L), and HbA1c (≥6.5 %) were used as the cutoff value for classifying T2DM in this study.

### Other measurements

BMI and waist–hip ratio (WHR) were assessed at baseline and follow-up using a standardized protocol as described elsewhere [33]. We collected self-reported information on medical history, family history in diabetes, medication use and lifestyle factors including physical activity [34]. Participants completed a validated semiquantitative FFQ [35] about their diet to assess average food intakes. Dietary intakes of total energy, total fatty acids, and specific fatty acids (En %) were calculated using an extended version of the Dutch Food Composition table from 2001 (NEVO) [36]. Enzymatic methods (Roche, Mannheim, Germany) were used to measure triglycerides and cholesterol values.

### Statistical analysis

Baseline characteristics of participants were presented in quartiles according to serum proportion of LA and ALA. To investigate the linear trend between serum LA or ALA and all baseline characteristics, we utilized a crude regression analysis with continuous measures.

After checking the data for potential effect modification by age, sex, use of statins, T2DM, or cohort origin (Hoorn study vs. Hoorn screening study), multiple linear regression tests were performed to investigate the cross-sectional association between baseline serum LA or ALA and baseline glucose parameters (FPG, PLG, and HbA1c). Adjustments were added in a stepwise approach, with Model III being the fully adjusted model that included adjustments for sex, age, total energy intake (kcal), BMI (kg/m^2^) and WHR (waist/hip ratio), physical activity (PA)(min/week), fiber (g/d), total dietary SFA (*E* %), alcohol (*E* %) and level of education. Additional confounding or mediation was addressed by stepwise adding other risk factors for T2DM: total cholesterol (mmol/L), use of statins (yes/no), or triglycerides (mmol/L).

Multiple linear regression tests were performed to investigate the association between serum LA and ALA proportions at baseline and FPG, PLG, and HbA1c at follow-up. We adjusted for the baseline levels of FPG, PLG, or HbA1c as well as for sex, age, total energy intake, and other risk factors similar to the models used in the cross-sectional analyses.

In addition to data on serum fatty acids proportions (as % of total fatty acids), absolute levels of serum fatty acids and data on dietary intake of LA and ALA via a FFQ were also available. To examine the validity of serum fatty acid proportions as markers of fatty acid intake, the Pearson correlation coefficients of total dietary LA and ALA with serum proportions and with absolute levels of LA or ALA were calculated. The associations between dietary intake of LA and ALA and the three blood glucose parameters were assessed with linear regression analyses.

SPSS version 18.0 was used for the statistical analyses (SPSS Inc. Released 2009. PASW Statistics for Windows, version 18.0. Chicago: SPSS Inc). A *p* value below 0.05 was considered statistically significant, except for interaction terms, where we used *p* < 0.10.

## Results

### Baseline characteristics

The mean age of the participants was 69 years (SD 7 years), and 49 % was male. In total, 128 (28 %) of the 667 study participants were newly diagnosed with T2DM. Mean proportions of serum LA and ALA were 26.98 % ± 3.81 and 0.53 % ± 0.15. The complete fatty acid profile of the population is shown in Supplementary Table 1. Characteristics of the study subjects across serum LA and ALA quartiles are displayed in Table 1. Individuals with higher serum LA proportions were more frequently male and had significantly lower BMI, WHR, dietary intake of SFA, triglycerides level, statins use and lower proportions of impaired fasting glucose (IFG)/impaired glucose tolerance (IGT), and T2DM. Also their levels of physical activity, LDL and HDL cholesterol levels were significantly higher, as well as their dietary intake of LA and ALA (%) and dietary fiber. Individuals with higher serum ALA proportions had significantly higher levels of physical activity and triglycerides, higher dietary ALA and fiber intake, and lower levels of HDL cholesterol.

**Table 1:** Baseline characteristics of 667 participants in the Hoorn study examination cycle 2000–2001 by quartiles (*Q*1–4) of serum LA and ALA

	LA									ALA								
	*Q*1 (*n* 166)		*Q*2 (*n* 167)		*Q*3 (*n* 167)		*Q*4 (*n* 167)		*p*	*Q*1 (*n* 166)		*Q*2 (*n* 167)		*Q*3 (*n* 167)		*Q*4 (*n* 167)		*p*
Range (% of total FA)	≤24.43		>24.43–26.96		>26.96–29.61		>29.61			≤0.43		>0.43–0.51		>0.51–0.60		>0.60		
Characteristics	Mean	SD	Mean	SD	Mean	SD	Mean	SD		Mean	SD	Mean	SD	Mean	SD	Mean	SD	
Age (years)	67.9	7.6	69.2	7.0	69.4	6.8	68.5	6.1	0.35	68.6	7.4	69.3	6.5	69.3	6.5	67.7	7.2	<.01*
Men (%)	51		41		46		57		0.06	43		48		49		56		<.01*
BMI (kg/m^2^)	28.7	4.1	28.1	4.9	27.0	3.6	26.4	3.8	<.001*	28.1	4.4	27.6	4.6	26.9	3.5	27.7	4.2	0.59
Waist–hip ratio	0.96	0.08	0.92	0.09	0.92	0.12	0.92	0.09	<.001*	0.93	0.08	0.93	0.12	0.92	0.09	0.94	0.09	0.12
Physical activity (min/w)	1271	927	1311	1062	1536	1153	1528	1004	<.01*	1343	974	1316	988	1342	956	1646	1215	0.02*
Triglycerides (mmol/L)	2.05	1.21	1.48	0.66	1.31	0.48	1.25	0.57	<.001*	1.29	0.62	1.44	0.72	1.49	0.80	1.86	1.05	<.001*
Systolic BP (mmHG)	141	18	143	20	141	21	140	22	0.45	143	20	140	21	140	20	142	21	0.32
Diastolic BP (mmHG)	85	11	83	12	82	11	82	10	0.03*	84	11	83	11	82	11	83	11	0.28
Total cholesterol (mmol/L)	5.65	1.07	5.64	1.00	5.74	1.08	5.73	1.03	0.77	5.61	1.08	5.79	1.07	5.65	1.01	5.70	1.01	0.41
LDL-c (mmol/L)	3.41	0.91	3.56	0.84	3.70	1.00	3.71	0.94	<.01*	3.56	1.00	3.69	0.93	3.58	0.88	3.56	0.91	0.58
HDL-c (mmol/L)	1.32	0.40	1.40	0.39	1.46	0.40	1.45	0.44	<.01*	1.47	0.41	1.46	0.42	1.40	0.41	1.31	0.40	<.001*
Statins (%)	23		20		14		7		<.001*	22		13		16		14		0.49
Total energy intake (kcal)	2013	617	1928	545	1987	832	1945	571	0.61	1874	953	2004	659	1936	578	1996	502	0.06
Dietary LA (*E* %)	4.68	1.68	5.17	1.67	5.55	1.58	6.10	1.82	<.001*	5.15	1.88	5.45	1.81	5.35	1.65	5.55	1.71	0.07
Dietary ALA (*E* %)	0.42	0.14	0.44	0.16	0.42	0.14	0.47	0.16	<.01*	0.42	0.15	0.42	0.14	0.44	0.14	0.47	0.17	<.001*
Dietary SFA (*E* %)	15.65	3.47	15.24	3.20	14.64	2.73	14.71	2.72	<.01*	15.04	3.19	14.94	3.32	15.04	3.00	15.21	2.76	0.55
Fiber (g/d)	20.8	6.2	21.7	5.9	22.9	6.1	23.7	6.7	<.001*	21.0	5.8	22.5	6.2	22.5	6.6	23.0	6.4	<.05*
Alcohol (*E* %)	6.49	7.68	4.05	4.82	2.89	4.19	3.59	4.21	<.001*	5.17	6.26	3.90	5.48	3.91	4.85	4.03	5.55	0.92
Prevalent CVD (%)	59		54		50		47		0.01*	51		58		55		47		0.26
Family history T2DM (%)	21		28		23		25		0.64	22		28		31		17		0.05
IFG/IGT (%)	33		25		27		23		<.001*	28		27		25		28		0.23
Newly diagnosed T2DM (%)	41		28		26		27		<.001*	40		25		27		30		0.08

Mean values and standard deviations (SD)

*LA* linoleic acid, *ALA* alpha-linolenic acid, *FA* fatty acids, *BP* blood pressure, *IFG* impaired fasting glucose, *IGT* impaired glucose tolerance, *T2DM* type 2 diabetes mellitus

Test for linear trend * *p* < 0.05

Mean levels of LA were associated with glucose tolerance state [27.7 % (3.6) for NGM, 26.5 % (3.8) for IFG/IGT, and 26.4 % (4.0) for T2DM, ANOVA *p* value <0.05]. Mean levels of ALA were not associated with glucose tolerance state [0.54 % (0.15) for NGM, 0.53 % (0.14) for IFG/IGT, and 0.52 % (0.17) for T2DM, ANOVA not significant].

### Cross-sectional analyses

Baseline serum LA proportions were inversely and significantly associated with FPG (*B* = −0.024 [−0.045, −0.002]) and PLG (*B* = −0.099 [−0.158, −0.039]) in the fully adjusted model. For PLG, this result remained significant after further adjustment for serum cholesterol and use of statins (*B* = −0.096 [−0.156, −0.035]) but not triglycerides. Significance was lost for FPG when additionally adjusted for cholesterol and statin use and triglycerides. There was no significant association between baseline serum LA proportions and baseline HbA1c (see Table 2). There was no significant association between serum ALA proportions and any of the three baseline blood glucose parameters. However, adjustment for triglycerides resulted in an inverse significant association of serum ALA with FPG (*B* = −0.810 [−1.333, −0.288]) and PLG (*B* = −2.335 [−3.837, −0.834]). There were no significant interactions between serum levels of LA or ALA and age, sex, use of statins, cohort origin, and T2DM (*p* > 0.10), except for the association between serum ALA and FPG, which was in the fully adjusted model stronger among subjects not taking statins (*p* value interaction = 0.052). Associations of serum polyunsaturated fatty acid levels other than LA and ALA are presented in Supplementary Table 2.

**Table 2:** Unstandardized regression coefficients of a cross-sectional linear regression analysis testing the association of serum LA and ALA proportions with three blood glucose parameters

	FPG mmol/L (*n* = 667)		PLG mmol/L (*n* = 537)		HbA1c (%) (*n* = 664)	
	*B* [95 % CI]		*B* [95 % CI]		*B* [95 % CI]	
LA (%)						
Model I	−0.054 [−0.077, −0.031]*		−0.130 [−0.186, −0.074]*		−0.006 [−0.019, 0.006]	
Model II	−0.027 [−0.050, −0.004]*		−0.091 [−0.148, −0.034]*		0.004 [−0.009, 0.017]	
Model III	−0.024 [−0.045, −0.002]*		−0.099 [−0.158, −0.039]*		0.000 [−0.014, 0.013]	
+ Cholesterol and statins^a^	−0.021 [−0.044, 0.001]		−0.096 [−0.156, −0.035]*		0.003 [−0.011, 0.017]	
+ Triglycerides^b^	−0.004 [−0.027, 0.019]		−0.054 [−0.117, 0.009]		0.012 [−0.003, 0.026]	
ALA (%)						
Model I	−0.534 [−1.111, 0.043]		−1.192 [−2.680, 0.296]		−0.076 [−0.395, 0.244]	
Model II	−0.508 [−1.056, 0.041]		−1.291 [−2.737, 0.154]		−0.088 [−0.404, 0.228]	
Model III	−0.292 [−0.794, 0.210]		−0.978 [−2.439, 0.482]		−0.057 [−0.372, 0.258]	
+ Cholesterol and statins^a^	−0.256 [−0.759, 0.247]		−0.894 [−2.366, 0.578]		−0.046 [−0.361, 0.268]	
+ Triglycerides^b^	−0.810 [−1.333, −0.288]*		−2.335 [−3.837, −0.834]*		−0.319 [−0.650, 0.012]	

Model I is adjusted for age, sex, and total energy intake

Model II is adjusted for age, sex, total energy intake, BMI, and WHR

Model III is adjusted for age, sex, total energy intake, BMI, WHR, physical activity, fiber, dietary SFA, alcohol, and education level

*FPG* fasting plasma glucose, *PLG* post-load glucose, *HbA1c* glycated hemoglobin, *B* unstandardized regression coefficient, *LA* linoleic acid, *ALA* alpha-linolenic acid

Linear regression analyses with * *p* < 0.05

^a^Stepwise adjustments (not cumulative) for cholesterol and statins to model III

^b^Stepwise adjustments (not cumulative) for triglycerides to model III

### Prospective analyses

After a mean follow-up time of 7.44 ± 0.55 year, 39 patients (20 male) out of 257 had developed T2DM. As shown in Table 3, baseline serum LA proportions were inversely and statistically significantly associated PLG (*B* = −0.112 [−0.187, −0.037]) at follow-up in Model I. However, this association was no longer statistically significant after adjustments for baseline PLG, and other covariates *B* = −0.026 [−0.100, 0.015], respectively). There was no association between baseline serum LA proportions and FPG or HbA1c levels at follow-up. Baseline serum ALA proportions were not significantly associated with FPG, PLG or HbA1c.

**Table 3:** Unstandardized regression coefficients of a prospective linear regression analysis testing the association of LA and ALA serum proportions at baseline with three blood glucose parameters at follow-up

	FPG mmol/L (*n* = 257)		PLG mmol/L (*n* = 253)		HbA1c (%) (*n* = 257)	
	*B* [95 % CI]		*B* [95 % CI]		*B* [95 % CI]	
LA (%)						
Model I	−0.025 [−0.050, 0.000]		−0.112 [−0.187, −0.037]*		−0.012 [−0.025, 0.001]	
Model II	−0.005 [−0.025, 0.016]		−0.054 [−0.123, 0.015]		−0.010 [−0.021, 0.001]	
Model III	0.003 [−0.019, 0.025]		−0.026 [−0.100, 0.049]		−0.007 [−0.019, 0.005]	
+ Cholesterol and statins^a^	0.004 [−0.019, 0.026]		−0.045 [−0.124, 0.033]		−0.002 [−0.015, 0.010]	
+ Triglyceride^b^	0.004 [−0.019, 0.026]		−0.045 [−0.124, 0.033]		−0.002 [−0.015, 0.010]	
ALA (%)						
Model I	−0.009 [−0.601, 0.584]		−1.616 [−3.426, 0.195]		0.043 [−0.260, 0.346]	
Model II	0.107 [−0.371, 0.585]		−1.240 [−2.853, 0.372]		0.065 [−0.198, 0.327]	
Model III	0.107 [−0.374, 0.588]		−1.098 [−2.716, 0.521]		0.072 [−0.191, 0.336]	
+ Cholesterol and statins^a^	0.114 [−0.375, 0.603]		−1.044 [−2.765, 0.677]		0.128 [−0.152, 0.408]	
+ Triglycerides^b^	0.114 [−0.375, 0.603]		−1.044 [−2.765, 0.677]		0.128 [−0.152, 0.408]	

Model I is adjusted for age, sex, and total energy intake

Model II is adjusted for age, sex, total energy intake, and baseline glycemic measure

Model III is adjusted for age, sex, total energy intake, baseline glycemic measure, BMI, WHR, physical activity, fiber, dietary SFA, alcohol, and education level

*FPG* fasting plasma glucose, *PLG* post-load glucose, *HbA1c* glycated hemoglobin, *B* unstandardized regression coefficient, *LA* linoleic acid, *ALA* alpha-linolenic acid

Linear regression analyses with * *p* < 0.05

^a^Stepwise adjustments (not cumulative) for cholesterol and statins to model III

^b^Stepwise adjustments (not cumulative) for triglycerides to model III

### Additional analyses

There was a significant correlation between dietary LA intake (*E* %) and serum proportions (% of total fatty acids) of LA (*r* = 0.343, *p* < 0.001) and a modest correlation with concentrations (micrograms/ml) of LA (*r* = 0.218, *p* < 0.001). There was a weaker correlation between dietary ALA intake and serum proportions of ALA (*r* = 0.134, *p* < 0.001) or concentrations (micrograms/ml) of ALA (*r* = 0.128, *p* < 0.001). Dietary LA intake was not significantly associated with any of the blood glucose parameters (*B* = 0.021 [−0.025, 0.068] for FPG, *B* = 0.082 [−0.045, 0.208] for PLG and *B* = 0.014 [−0.016, 0.043] for HbA1c. The same was true for dietary ALA intake (*B* = 0.249 [−0.275, 0.773] for FPG, *B* = 0.937 [−0.520, 2.40] for PLG and *B* = 0.196 [−0.131, 0.524] for HbA1c).

## Discussion and conclusion

In the present population-based cross-sectional study, we observed significant inverse associations of LA in serum with concentrations of FPG and PLG, but not with HbA1c. The proportion of ALA in serum was not associated with these markers of glucose metabolism. These associations were independent of age, sex, total energy intake, obesity, and other lifestyle factors. In prospective analyses of this same population, however, there were no significant independent associations between serum LA and ALA proportions and blood glucose concentrations.

### Strengths and limitations

A major strength of this study is the measurement of the proportions of LA and ALA in serum as markers of their dietary intake. Intake of fatty acids can be difficult to estimate accurately from FFQ because of misreporting by subjects and lack of data on fatty acid content in food composition tables [7]. As a result, important associations between fatty acid intake and health outcomes can be missed when only assessed with dietary fat intake from a FFQ [18]. This is supported by the current study, in which we found an inverse cross-sectional association between serum LA and glucose concentration, while this was not observed for dietary LA.

Total fatty acid levels in serum, as measured in the present study, originate from different blood lipid fractions including triglycerides, cholesteryl esters, phospholipids, and nonesterified fatty acids. These lipid fractions reflect dietary intake in previous days to weeks at maximum [18]. Short-term dietary modifications reflected in serum fatty acids may not translate into changes in risk factors within the same timeframe, thereby potentially underestimating the true association. Nevertheless, serum LA and ALA levels do reasonably well correlate with dietary LA and ALA intake, as assessed via FFQ (typically reflecting intake over previous 4 weeks) [18].

An advantage of the current study is that we determined FPG, PLG, as well as HbA1c, three markers of different aspects of glucose metabolism, that are each considered relevant and are accepted for the diagnosis of T2DM [32]. This provides the opportunity to investigate the associations of serum and dietary LA and ALA with different aspects of the glucose metabolism. Other strengths of the current study include the population-based prospective design and the detailed characteristics of the study population.

Several limitations of our analysis are to be addressed. It might be argued that the overrepresentation of T2DM patients and persons with impaired glucose tolerance in our population limits the generalizability of our findings to other populations. On the other hand, the advantage of a population with highly prevalent T2DM and impaired glucose tolerance is that this enhances the likelihood to detect larger differences in glucose metabolism between subjects. Regardless, the current study and in particular the prospective analyses are limited by sample size and may thus lack power to assess a potential association with diabetes incidence. Serum fatty acid proportions and glucose parameters can be influenced by age, sex, total energy intake, obesity, lifestyle factors, and other (unknown) confounders. Despite careful adjustments for most of these variables, we cannot rule out residual or unmeasured confounding. There is also the chance that our results were over-adjusted by including waist–hip ratio as potential confounder in the model. There are indications that PUFA, in comparison with SFA, may reduce abdominal fat [37], which could lead to a reduction in blood glucose. The waist–hip ratio reflecting abdominal fat could thus be in the causal pathway from dietary and serum LA to T2DM, in which case the adjustment for waist–hip ratio would underestimate their relationship in the population. Another study limitation is that the proportions of LA and ALA in serum were assessed only once at baseline. Potential changes in intake of LA and ALA over time could have caused misclassification of long-term dietary exposure and in this way underestimation of the underlying association.

### Current findings in the context of other studies

As in most epidemiological studies, serum fatty acid levels in the current study were measured and expressed as proportion of total fatty acids. We observed that the correlation of dietary LA and ALA intake with serum proportions of LA and ALA is stronger than the correlation with concentrations of LA and ALA (present study). This may favor the expression of LA and ALA as a proportion of total fatty acids.

We found that higher proportions of serum LA in cross-sectional analyses were associated with lower levels of blood glucose concentrations. These findings are in line with results from earlier prospective cohort studies on the relationship between serum LA and T2DM incidence [13, 14, 19–23]. Our findings are also largely in line with results from a recent systematic review, which concluded that higher serum levels of ALA tend to be associated with lower risk of T2DM [24]. LA or ALA proportions in serum did not show an association with HbA1c. This may suggest that for HbA1c physiological and pathological determinants other than diet are more important than for glucose [29].

There was a weak correlation between serum ALA and markers of blood glucose metabolism in our study, which has also been observed in other studies [38]. It has been suggested to be the result of rapid oxidation of dietary ALA in the body [39] or of measurement error in the intake data including underlying food composition tables [38].

### Potential mechanisms

Biological mechanisms underlying a possible relation between dietary and serum fatty acids and risk of T2DM are not completely understood. It has been hypothesized that consuming a diet with a high ratio of PUFA to SFA increases the PUFA content of phospholipids, which may improve the glucose transport, oxidation, and lipogenesis [40]. The ratio of PUFA to SFA may influence the flexibility of the cell membrane, cellular function, and insulin signaling leading to an increase in insulin sensitivity when more PUFA is consumed. Indeed, one intervention trial in which PUFA was replaced by SFA reported a beneficial effect on insulin sensitivity [41]. In the present study, serum LA proportions showed a significant inverse association with both FPG and PLG. This is plausible given that FPG and PLG are the primary markers of insulin sensitivity in the liver and the peripheral tissues, respectively [42]. Part of the observed association between LA proportions and T2DM risk in our study was explained by differences in triglyceride levels. Dyslipidemia, involving elevated triglyceride levels, is a common characteristic in patients with T2DM [43]. In addition, since the proportion of LA varies between lipid species, the lipid levels may have confounded the relationship between LA and glucose. In cholesteryl esters, LA compose over half of fatty acids, whereas in triglycerides the proportion of LA is about 15 % and in phospholipids about 20 %. This implies that among individuals with higher concentrations of triglycerides, the proportion of LA in total serum is lower [18]. However, the causality of the relationship between LA proportion and lipid species is poorly understood. One might suggest that increases in triglycerides lead to a reduction in LA levels. On the other hand, changes in triglycerides, and its associated etiologies insulin resistance and ectopic fat deposition may be the result of dietary LA intake. The latter is supported by controlled human dietary intervention studies, in which PUFA as replacement of SFA reduced liver fat [44, 37] and insulin resistance [41]. In addition, a meta-analysis of 60 controlled intervention studies on blood lipids has established that dietary PUFA intake reduce triglyceride levels compared with carbohydrates [1]. Thus, adjustment for triglycerides in our models will correct for variation in LA between lipid species, but may lead to underestimation of the true association because it partially corrects for triglycerides as an intermediate in the causal pathway toward higher glucose levels. Therefore, we assume that true association between LA and glucose levels may lie in between the associations estimated by the triglyceride-adjusted and unadjusted models.

The proportion of LA in serum fatty acid is not solely influenced by LA intake, but also by endogenous fatty acid metabolism [38]. For example, an increased Δ6-desaturase activity, resulting in lower serum LA, has been associated with insulin resistance and T2DM [45, 46]. Therefore, reversed causality cannot be excluded as an explanation for the observed association. For the present study, however, this is not very likely given that Δ6-desaturase activity (estimated as C18:3n6/C18:2n6 ratio) in T2DM subjects was not significantly different from nondiabetic subjects (data not shown). The biological mechanisms by which different types of PUFAs might affect glucose metabolism remain to be elucidated.

## Conclusion

In this population with overrepresentation of subjects with glucose intolerance, serum LA was inversely associated with fasting and post-load glucose in cross-sectional, but not in prospective analyses. Further studies are needed to elucidate the potential role of serum LA and ALA levels and dietary PUFA in glucose metabolism.

## Electronic supplementary material

Below is the link to the electronic supplementary material.

Supplementary material 1 (DOCX 20 kb)
