# Supplementary material for: Circulating linoleic acid and alpha-linolenic acid and glucose metabolism: the Hoorn Study
Source: Eur J Nutr. 2016 Jul 14;56(6):2171–80. doi: 10.1007/s00394-016-1261-6 (PMC5579177; doi:10.1007/s00394-016-1261-6)
Supplement: Supplementary file 1 — Supplementary material 1 (DOCX 20 kb) [file 394_2016_1261_MOESM1_ESM.docx]

# Supplementary Table 1

Fatty acid composition measured in total plasma fatty acids in the Hoorn study (n=667).

| Variable | Name | Mean (%) | SD |
| --- | --- | --- | --- |
| **Total SFA** |  | 32.83 | 1.71 |
| C14:0 | Myristic Acid | 1.17 | 0.37 |
| C15:0 | Pentadecyclic Acid | 0.22 | 0.05 |
| C16:0 | Palmitic Acid | 22.48 | 1.64 |
| C18:0 | Stearic Acid | 6.89 | 0.57 |
| C20:0 | [Arachidic Acid](http://en.wikipedia.org/wiki/Arachidic_acid) | 0.26 | 0.05 |
| C22:0 | [Behenic Acid](http://en.wikipedia.org/wiki/Behenic_acid) | 0.73 | 0.16 |
| C23:0 | [Tricosylic Acid](http://en.wikipedia.org/w/index.php?title=Tricosylic_acid&action=edit&redlink=1) | 0.31 | 0.07 |
| C24:0 | [Lignoceric Acid](http://en.wikipedia.org/wiki/Lignoceric_acid) | 0.76 | 0.18 |
| **Total TFA** |  | 0.86 | 0.26 |
| **Total MUFA** |  | 23.11 | 2.79 |
| C16:1n7 | Palmitoleic Acid | 2.38 | 0.78 |
| C18:1n9 | Oleic Acid | 18.05 | 2.35 |
| C18:1n7 | Cis-Vaccenic Acid | 1.43 | 0.22 |
| C20:1n9 | Eicosenoic Acid | 0.12 | 0.04 |
| C24:1n9 | Nervonic Acid | 1.13 | 0.27 |
| **Total PUFA** |  | 37.76 | 3.97 |
| Total n-6 |  | 34.13 | 3.92 |
| C18:2n6 | Linoleic Acid | 26.98 | 3.81 |
| C18:3n6 | Gamma linolenic Acid | 0.37 | 0.13 |
| C20:2n6 | Eicosadienoic Acid | 0.19 | 0.04 |
| C20:3n6 | Dihomo-γ-linolenic Acid | 1.39 | 0.29 |
| C20:4n6 | Arachidonic Acid | 5.21 | 1.19 |
| Total n-3 |  | 3.63 | 1.28 |
| C18:3n3 | Alpha linolenic Acid | 0.53 | 0.15 |
| C20:5n3 | Eicosapentaenoic Acid | 0.83 | 0.58 |
| C22:5n3 | Docosapentaenoic Acid | 0.48 | 0.11 |
| C22:6n3 | Docosahexaenoic Acid | 1.79 | 0.67 |
|  |  |  |  |

FPG, fasting plasma glucose; PLG, post load glucose; HbA1c, glycated hemoglobin; *B*, unstandardized regression coefficient; SFA, saturated fatty acids; TFA, trans fatty acids includes only C18:1t6-11; MUFA, monounsaturated fatty acids; PUFA, polyunsaturated fatty acids;

# Supplementary Table 2

Regression Coefficients of Cross-sectional Linear Regression Analyses† testing the Association of Serum Polyunsaturated Fatty acids and Enzymatic Conversion Activities with Blood Glucose Parameters in the Hoorn study (n=667).

|  |  | FPG | PLG | HbA1c |
| --- | --- | --- | --- | --- |
| Variable | Name | *B* [95% CI] | *B* [95% CI] | *B* [95% CI] |
| **Total PUFA** |  | -0.030 [-0.050, -0.009]* | -0.105 [-0.161, -0.049]* | -0.006 [-0.016, 0.007] |
| **Total n-6** |  | -0.024 [-0.045, -0.003]* | -0.086 [-0.144, -0.028]* | -0.006 [-0.019, 0.007] |
| C18:2n6 | Linoleic Acid | -0.024 [-0.045, -0.002]* | -0.099 [-0.158, -0.039]* | 0.000 [-0.014, 0.013] |
| C18:3n6 | Gamma linolenic Acid | 0.097 [-0.526, 0.720] | 2.207 [0.533, 3.882]* | -0.162[-0.552, 0.227] |
| C20:2n6 | Eicosadienoic Acid | 1.284 [-0.896, 3.464] | 5.030 [-0.896, 10.956] | 0.769 [-0.596, 2.133] |
| C20:3n6 | Dihomo-γ-linolenic Acid | 0.151 [-0.120, 0.423] | 0.950 [0.205, 1.696]* | -0.121 [-0.290, 0.050] |
| C20:4n6 | Arachidonic Acid | -0.037 [-0.102, 0.028] | -0.030 [-0.209, 0.149] | -0.045 [-0.085, -0.004]* |
| **Total n-3** |  | -0.055 [-0.116, 0.005] | -0.202 [-0.364, -0.041]* | -0.001 [-0.039, 0.037] |
| C18:3n3 | Alpha linolenic Acid | -0.292 [-0.794, 0.210] | -0.978 [-2.439, 0.482] | -0.057 [-0.372, 0.258] |
| C20:5n3 | Eicosapentaenoic Acid | -0.128 [-0.261, 0.006] | -0.471 [-0.827, -0.116]* | 0.001 [-0.083, 0.085] |
| C22:5n3 | Docosapentaenoic Acid | -1.220 [-1.925, -0.515]* | -1.348 [-3.280, 0.584] | -0.501 [-0.945, -0.057]* |
| C22:6n3 | Docosahexaenoic Acid | -0.058 [-0.174, 0.058] | -0.300 [-0.607, 0.007] | 0.012 [-0.061, 0.084] |
| **Desaturases** |  |  |  |  |
| C18:3n6/C18:2n6 | Δ6 desaturase | 10.4 [-3.5, 24.3] | 66.9 [30.2, 103.7]* | -0.6 [-9.3, 8.2] |
| C20:4n6/C20:3n6 | Δ5 desaturase | -0.055 [-0.123, 0.012] | -0.192 [-0.375, -0.009]* | -0.018 [-0.060, 0.024] |
| **Elongases** |  |  |  |  |
| C20:3n6/C18:3n6 |  | 0.004 [-0.052, 0.060] | -0.083 [-0.232, 0.066] | 0.003 [-0.032, 0.038] |
| C22:5n3/C20:5n3 |  | 0.215 [-0.045, 0.475] | 0.827 [0.060, 1.594]* | -0.080 [-0.243, 0.083] |

FPG, fasting plasma glucose; PLG, post load glucose; HbA1c, glycated hemoglobin; *B*, unstandardized regression coefficient; PUFA, polyunsaturated fatty acids;

*Statistically significant at P<.05

†The models were adjusted for age, gender, total energy intake, BMI, WHR, physical activity, fiber, dietary SFA, alcohol and education level
